# Sterile leukocyturia − Difficult diagnostic problem in children

**DOI:** 10.34763/devperiodmed.20172102.139143

**Published:** 2017-08-11

**Authors:** Agnieszka Szmigielska, Grażyna Krzemień

**Affiliations:** 1 Katedra i Klinika Pediatrii i Nefrologii, Warszawski Uniwersytet Medyczny, Polska

**Keywords:** leukocyturia, jałowa leukocyturia, badanie ogólne moczu, atypowe zakażenie układu moczowego, dzieci, leukocyturia, sterile leukocyturia, urinalysis, atypical urinary tract infection, children

## Abstract

Sterile leukocyturia is an important and difficult clinical problem in children. In this paper, we described the most common nephrologic causes of sterile leukocyturia, including infectious, non-infectious and extrarenal etiology. We stressed an the importance of appropriate urine collection for urinalysis. There is a need for treatment of inflammation and also for diagnosis of potential anomalies of urethral orifice as causes of sterile leukocyturia in children.

## Streszczenie

Jałowa leukocyturia stanowi istotny i trudny problem kliniczny u dzieci. W artykule omówiono najczęstsze nerkowe przyczyny jałowej leukocyturii o podłożu infekcyjnym i nieinfekcyjnym oraz przyczyny pozanerkowe. Podkreślono znaczenie prawidłowego pobrania moczu na badanie ogólne oraz wyeliminowania stanu zapalnego i nieprawidłowości okolicy cewki moczowej jako przyczyny jałowej leukocyturii u dzieci.

## Wstęp

W warunkach prawidłowych w badaniu ogólnym moczu mogą być obecne pojedyncze krwinki białe. Leukocyturia oznacza obecność w moczu nieodwirowanym ponad 5 leukocytów w polu widzenia (wpw) mikroskopu (powiększenie 400 razy) lub powyżej 10 leukocytów wpw w moczu odwirowanym [1]. W przypadku znacznie nasilonej leukocyturii używa się określenia ropomocz. U dzieci powyżej 2. roku życia w rozpoznawaniu leukocyturii mogą być przydatne testy paskowe oceniające obecność esterazy leukocytów. Czułość i swoistość testu na aktywność esterazy leukocytów wynosi odpowiednio 83% i 78%. Obecność witaminy C w moczu może być przyczyną fałszywie ujemnego wyniku testu [2]. Najczęstszą przyczyną leukocyturii u dzieci jest zakażenie układu moczowego (ZUM) o etiologii bakteryjnej, rozpoznawane na podstawie wzrostu z posiewu moczu bakterii w liczbie znamiennej i leukocyturii [1]. Prawidłowe badanie ogólne moczu pozwala u większości dzieci wykluczyć ZUM. Czasami badanie ogólne moczu wykonane we wczesnej fazie zakażenia jest prawidłowe. Ma to związek z opóźnioną reakcją zapalną ustroju na zakażenie. Dlatego u dzieci z gorączką utrzymującą się bez uchwytnej przyczyny konieczne jest powtórzenie badania ogólnego moczu [3]. U około 1-3% dzieci stwierdza się wzrost w moczu bakterii w liczbie znamiennej przy prawidłowym badaniu ogólnym moczu i braku objawów klinicznych ZUM [1]. Jest to tzw. bezobjawowa bakteriuria, która w większości przypadków ustępuje samoistnie i nie wymaga leczenia. [3]. Jałowa leukocyturia oznacza obecność krwinek białych w badaniu ogólnym moczu przy ujemnym wyniku badania bakteriologicznego moczu. W różnicowaniu przyczyn jałowej leukocyturii należy uwzględnić szereg czynników, co sprawia, że diagnostyka nie jest łatwa.

## Przyczyny jałowej leukocyturii

### 1 Nieprawidłowy sposób pobrania moczu na badanie ogólne

Najczęstszą przyczyna jałowej leukocyturii u dzieci, bez objawów klinicznych ZUM, jest nieprawidłowy sposób pobrania moczu na badanie ogólne. Przed pobraniem moczu do badania należy dokładnie umyć krocze z odsłonięciem napletka u chłopców, u dziewczynek należy rozchylić wargi sromowe. Próbka moczu na badanie ogólne może być pobrana ze środkowego strumienia moczu podczas mikcji lub z jałowego woreczka przyklejonego do krocza. Woreczek powinien być odklejony natychmiast po oddaniu moczu, a mocz dostarczony do laboratorium w ciągu 1-2 godzin [1]. Obecność leukocyturii w moczu pobranym do woreczka wymaga powtórzenia badania ze środkowego strumienia. Nie należy pobierać do woreczka moczu na badanie bakteriologiczne [1].

### 2 Stany zapalne lub nieprawidłowości okolicy cewki moczowej

Obecność jałowej leukocyturii, szczególnie u niemowląt, może wynikać nie tylko ze złej techniki pobierania moczu na badanie, ale być następstwem stanu zapalnego okolicy krocza, zlepu warg sromowych (synechii), stulejki lub współistniejącego nieżytu żołądkowo-jelitowego [3]. U noworodków i niemowląt leukocyturia może być wynikiem przedostania się do próbki moczu wydzieliny z pochwy. Przemawia za tym obecność śluzu w moczu.

### 3 „Fałszywie” ujemna jałowa leukocyturia

Należy podkreślić, że nawet jedna dawka antybiotyku lub chemioterapeutyku podana przed pobraniem moczu na posiew może zahamować wzrost bakterii w moczu i być przyczyną jałowej leukocyturii u dziecka z ZUM. Inną przyczyną jałowej leukocyturii może być skrócenie czasu namnażania bakterii w układzie moczowym z powodu częstomoczu lub wielomoczu. Częstomocz najczęściej jest objawem zapalenia lub złogu zlokalizowanego w pęcherzu lub cewce moczowej. Wielomocz może być objawem wielu chorób. Obserwowany jest u dzieci z cukrzycą, we wstępnej fazie przewlekłej choroby nerek, w moczówce centralnej i nerkowej, nefronoftyzie, w niektórych tubulopatiach np. w zespole Fanconiego. Może być objawem hipokaliemii i hiperkalcemii niezależnie od ich przyczyny [3].

### 4 Atypowe zakażenia układu moczowego

U dzieci z utrzymującą się jałową leukocyturią, u których mocz na badanie ogólne został prawidłowo pobrany, nie podano leków przeciwbakteryjnych przed pobraniem posiewu moczu oraz wykluczono częstomocz i wielomocz należy rozszerzyć badania diagnostyczne w kierunku atypowych zakażeń układu moczowego.

#### a) Wirusowe

Zakażenie układu moczowego o etiologii wirusowej najczęściej występuje jako zapalenie pęcherza moczowego i cewki moczowej z krwinkomoczem lub krwiomoczem, rzadziej z jałową leukocyturią. Wirusowe ZUM występuje głównie u osób z pierwotnymi lub wtórnymi zaburzeniami odporności. Przyczyną wirusowego ZUM u dzieci są głównie wirusy *Herpes simplex* i adenowirusy wywołujące krwotoczne zapalenie pęcherza moczowego, rzadziej wirusy *HPV* [4, 5]. Po transplantacji nerki lub przeszczepieniu szpiku zakażenie może być wywołane przez wirusa cytomegalii lub polioma BK [6].

#### b) Grzybicze

Zakażenie układu moczowego o etiologii grzybiczej występuje głównie u dzieci z upośledzoną odpornością, długotrwale leczonych antybiotykami i lekami immunosupresyjnymi, urodzonych przedwcześnie lub chorujących na cukrzycę. Może być powikłaniem wad utrudniających odpływ moczu, kamicy układu moczowego i instrumentacji dróg moczowych. Zakażenie o etiologii grzybiczej należy podejrzewać u każdego dziecka z jałową leukocyturią i objawami ZUM, u którego stwierdza się czynniki ryzyka wystąpienia grzybiczego ZUM. Najczęstszą przyczyną zakażenia są grzyby z rodzaju *Candida: Candida albicans, Candida glabrata, Candida krusei*, a źródłem zakażenia przewód pokarmowy. Rzadziej izoluje się z moczu inne gatunki grzybów np. *Cryptococcus neoformans* i *Aspergillus sp*. [7, 8, 9]. Grzybicze zakażenie może prowadzić do tworzenia grzybniaków w układzie moczowym i zaburzeń w odpływie moczu. W badaniu USG można stwierdzić w układzie kielichowo-miedniczkowym hiperechogenne odbicia bez stożka cienia, które odpowiadają grzybniakom. W ocenie rozległości zmian grzybiczych przydatna jest tomografia komputerowa [10].

#### c) Gruźlicze

Po limfadenopatii, układ moczowy jest jedną z najczęstszych pozapłucnych lokalizacji gruźlicy. Zakażenie gruźlicze jest trudne do rozpoznania, średni czas od wystąpienia pierwszych objawów klinicznych do rozpoznania wynosi 8-9 miesięcy. Do najczęstszych objawów zakażenia należą: krwinkomocz (79,7%), jałowa leukocyturia (67,1%) i objawy dyzuryczne (51,9%) [11, 12]. Niska wykrywalność gruźliczego ZUM jest związana z koniecznością zastosowania odpowiednich podłoży do hodowli prątków i długim oczekiwaniem na wynik badania bakteriologicznego (nawet do 8 tygodni). Wzrost *Mycobacterium tuberculosis* na podłożu standardowym jest widoczny tylko u około 37% chorych. Badanie moczu metodą PCR pozwala potwierdzić gruźlicze zakażenie u ponad 94% chorych [13]. W diagnostyce zakażenia o tej etiologii przydatne jest badanie USG i tomografia komputerowa, które mogą uwidocznić zwapnienia w nerkach, wodonercze lub blizny w miąższu nerek. W badaniach obrazowych zmiany gruźlicze w układzie moczowym widoczne są w chwili pojawienia się zwapnień w ogniskach serowaciejących lub nadżerek w sklepieniach kielichów. W przypadku długotrwałej gruźlicy widoczne są wielopoziomowe zwężenia w układzie moczowym [10].

#### d) Przenoszone najczęściej drogą płciową

Jałowa leukocyturia u nastolatków może być objawem choroby przenoszonej drogą płciową (STD – sexually transmitted disease). Do najczęstszych czynników etiologicznych STD należą: *Neisseria gonorrhoeae, Chlamydia*

*trachomatis, Ureaplasma urealyticum, Mycoplasma hominis, Mycoplasma genitalium*. Według Centers for Disease Control and Prevention (CDC) w Stanach Zjednoczonych co roku stwierdza się około 300 000 zakażeń wywołanych przez *Neisseria gonorrhoeae*. Do zakażeń powyższymi patogenami może dojść nie tylko drogą płciową, ale również przez bezpośredni kontakt z zakażoną skórą okolicy narządów płciowych. Drobnoustroje te wywołują zapalenie pęcherza moczowego i cewki moczowej. Objawy kliniczne zakażenia częściej stwierdza się u chłopców, u dziewczynek przebieg zakażenia może być bezobjawowy [14, 15]. Nieleczone STD mogą być przyczyną wielu groźnych powikłań narządowych lub być przyczyną niepłodności.

#### e) Pasożytnicze

W różnicowaniu przyczyn jałowej leukocyturii należy brać pod uwagę także zakażenia pasożytnicze wywołane przez *Trichomonas vaginalis* i *Schistosoma haematobium*. Zakażenie *Trichomonas vaginalis* jest najczęstszą przyczyną infekcji pasożytniczych w Stanach Zjednoczonych. Zakażenie narządów płciowych może być przyczyną stwierdzanej w tym przypadku jałowej leukocyturii. Obecność pasożyta w moczu można stwierdzić w badaniu mikroskopowym lub potwierdzić metodą PCR. Do zakażenia *Schistosoma haematobium* u większości osób dochodzi w czasie pobytu w Afryce, co należy uwzględnić w wywiadzie chorobowym. W ZUM o tej etiologii stwierdza się charakterystyczne zmiany w badaniach obrazowych tzn. kalcyfikację ściany pęcherza i moczowodów [16, 17].

### 5 Nieinfekcyjne choroby układu moczowego

#### a) Ostre i przewlekłe kłębuszkowe zapalenia nerek

Jałowa leukocyturia najczęściej jest obserwowana u dzieci z ostrym kłębuszkowym zapaleniem nerek (OKZN), które jest główną przyczyną zespołu nefrytycznego w krajach rozwijających się. W krajach rozwiniętych częstość zachorowań na OKZN wynosi 0,3:100 000 populacji i ulega systematycznemu obniżaniu w związku z większą skutecznością leczenia zakażeń paciorkowcowych u dzieci. Objawy kliniczne w OKZN mają różne nasilenie, od niewielkiego krwinkomoczu do pełnoobjawowego zespołu nefrytycznego (białkomoczu nienerczycowego, krwinkomoczu, nadciśnienia tętniczego i uszkodzenia nerek). Występowanie jałowej leukocyturii jest związane z odczynem zapalnym w kłębuszkach nerkowych i tkance śródmiąższowej [21]. Jałową leukocyturię stwierdza się w chorobach układowych z zajęciem nerek, najczęściej w toczniu rumieniowatym układowym i w zapaleniach naczyń np. w chorobie Kawasakiego [22, 23].

#### b) Nieinfekcyjne ostre cewkowo-śródmiąższowe zapalenie nerek

Do wystąpienia ostrego cewkowo-śródmiąższowego zapalenia nerek (OCŚZN) może dojść w następstwie stosowania różnych leków, najczęściej antybiotyków (penicyliny, cefalosporyny, wankomycyna, erytromycyna, tetracykliny, rifampicyna), sulfonamidów, niesteroidowych leków przeciwzapalnych, leków moczopędnych i przeciwdrgawkowych. W OCŚZN naciek zapalny pierwotnie dotyczy tkanki śródmiąższowej i cewek nerkowych. Zwykle powoduje gwałtowne zaburzenie czynności nerek [10]. Do typowych objawów choroby należą gorączka, wysypka, bóle stawów, eozynofilia, eozynofiluria, białkomocz, krwinkomocz i jałowa leukocyturia [18, 19, 20]. U około 7% dzieci OCŚZN jest przyczyną ostrej niewydolności nerek [21].

#### c) Kamica układu moczowego

Kamica układu moczowego u dzieci rozpoznawana jest z częstością 10-15%. [24]. Najczęstszym objawem kamicy jest krwinkomocz, krwiomocz i kolka nerkowa. Jałowa leukocyturia może pojawić się w wyniku odczynu zapalnego na obecność złogu w miedniczce nerkowej lub moczowodzie. Większość złogów w drogach moczowych wykrywana jest w badaniu USG. Zdjęcie przeglądowe jamy brzusznej i urografia u dzieci są obecnie rzadziej wykonywane. Największą czułość w wykrywaniu złogów w drogach moczowych wykazuje tomografia komputerowa. Czynnikami ryzyka wystąpienia kamicy jest nieodpowiednia dieta i obciążony wywiad rodzinny. U dzieci istotną rolę w rozwoju kamicy odgrywają zaburzenia metaboliczne – najczęściej hiperkalciuria, hiperoksaluria, hiperurikozuria i hipocytraturia. Kamica tzw. infekcyjna jest związana z zakażeniem bakteriami wytwarzającymi ureazę (głównie *Proteus, Pseudomonas, Klebsiella*) i występuje stosunkowo rzadko. U każdego dziecka z kamicą układu moczowego należy przeprowadzić wnikliwą diagnostykę w celu ustalenia przyczyny kamicy i wdrożenia odpowiedniego postępowania [25].

#### e) Nowotwory układu moczowego

Najczęstszym nowotworem nerki u dzieci jest nefroblastoma – guz Wilmsa, który stanowi 6-9% wszystkich nowotworów wieku dziecięcego. Nowotwór zwykle rozpoznawany jest przypadkowo, gdy po osiągnięciu znacznych rozmiarów powiększa obwód brzucha. U 15% dzieci z guzem Wilmsa stwierdza się krwinkomocz lub krwiomocz, a u około 3% dzieci zakażenie układu moczowego. Najczęstszym nowotworem pęcherza moczowego u dzieci jest mięsak prążkowano komórkowy [26]. Jałowa leukocyturia jest objawem odczynu zapalnego na obecność nowotworu w tkance śródmiąższowej lub drogach moczowych [27].

#### f) Wady układu moczowego i zabiegi urologiczne

Jałowa leukocyturia jest obserwowana u dzieci z wadami układu moczowego utrudniającymi odpływ moczu, z drenażem górnych dróg moczowych lub pęcherza moczowego oraz po zabiegach urologicznych. W każdym przypadku u dziecka z jałową leukocyturią i obciążonym wywiadem urologicznym należy wykluczyć atypowe ZUM.

### 6 Przyczyny pozanerkowe

#### a) Stany zapalne układu pokarmowego i rozrodczego

Jałowa leukocyturia występuje u dzieci z nieswoistymi zapaleniami jelit (choroba Leśniowskiego-Crohna, wrzodziejące zapalenie jelit), u 87,5% chorych z zapaleniem wyrostka robaczkowego, u 72,7% z zapaleniem uchyłków jelita grubego, z ropniami okołonerkowymi i międzypętlowym [28, 29, 30]. Może towarzyszyć zapaleniom przydatków u dziewczynek i stanom zapalnym pochwy. U małych dzieci należy wykluczyć obecność ciała obcego w pochwie.

#### b) Inne

Jałowa leukocyturia i białkomocz jest obserwowana u pacjentów z niewydolnością krążenia, w stanach znacznego odwodnienia, u wysoko gorączkujących dzieci z powodu różnych infekcji zlokalizowanych poza układem moczowym oraz u zdrowych osób po znacznym wysiłku fizycznym [27, 231]. U każdego dziecka z gorączką i leukocyturią należy wykluczyć ZUM. W przypadku odwodnienia i współistniejącej leukocyturii, po nawodnieniu pacjenta należy niezwłocznie powtórzyć badanie ogólne moczu w celu wykluczenia zakażenia.

## Podsumowanie

1. Różnicowanie jałowej leukocyturii należy rozpocząć od zebrania wywiadu uwzględniającego: sposób pobrania moczu do badania, objawy kliniczne obserwowane u dziecka, przyjmowane leki, wywiad rodzinny w kierunku chorób nerek, kontakt z osobami chorymi na gruźlicę, aktywność seksualną oraz podróże do krajów egzotycznych.
2. W badaniu przedmiotowym należy dokładnie obejrzeć okolicę krocza wykluczając częste przyczyny leukocyturii takie jak zlep warg sromowych u dziewczynek, stulejkę u chłopców i stany zapalne krocza.
3. W przypadku stwierdzenia jałowej leukocyturii antybiotykoterapię stosuje się tylko w wyjątkowych przypadkach np. chorobach przenoszonych drogą płciową.
